# Development and validation of a predictive model based on clinical and MpMRI findings to reduce additional systematic prostate biopsy

**DOI:** 10.1186/s13244-023-01544-0

**Published:** 2024-01-07

**Authors:** Xueqing Cheng, Yuntian Chen, Jinshun Xu, Diming Cai, Zhenhua Liu, Hao Zeng, Jin Yao, Bin Song

**Affiliations:** 1 Department of Radiology, West China Hospital of Sichuan University, No. 37 Guoxue Street, Chengdu, 610041 Sichuan China; 2 Functional and Molecular Imaging Key Laboratory of Sichuan Province, West China Hospital of Sichuan University, Chengdu, Sichuan China; 3 Department of Ultrasound, Sichuan Cancer Hospital, Chengdu, Sichuan China; 4 Department of Ultrasound, West China Hospital of Sichuan University, Chengdu, Sichuan China; 5 Department of Urology, West China Hospital of Sichuan University, Chengdu, Sichuan China; 6 Department of Radiology, Sanya People’s Hospital, Sanya, Hainan China

**Keywords:** Prostate cancer, Magnetic resonance imaging, Nomogram, Biopsy

## Abstract

**Objectives:**

To develop and validate a predictive model based on clinical features and multiparametric magnetic resonance imaging (mpMRI) to reduce unnecessary systematic biopsies (SBs) in biopsy-naïve patients with suspected prostate cancer (PCa).

**Methods:**

A total of 274 patients who underwent combined cognitive MRI-targeted biopsy (MRTB) with SB were retrospectively enrolled and temporally split into development (*n* = 201) and validation (*n* = 73) cohorts. Multivariable logistic regression analyses were used to determine independent predictors of clinically significant PCa (csPCa) on cognitive MRTB, and the clinical, MRI, and combined models were established respectively. Area under the receiver operating characteristic curve (AUC), calibration plots, and decision curve analyses were assessed.

**Results:**

Prostate imaging data and reporting system (PI-RADS) score, index lesion (IL) on the peripheral zone, age, and prostate-specific antigen density (PSAD) were independent predictors and included in the combined model. The combined model achieved the best discrimination (AUC 0.88) as compared to both the MRI model incorporated by PI-RADS score, IL level, and zone (AUC 0.86) and the clinical model incorporated by age and PSAD (AUC 0.70). The combined model also showed good calibration and enabled great net benefit. Applying the combined model as a reference for performing MRTB alone with a cutoff of 60% would reduce 43.8% of additional SB, while missing 2.9% csPCa.

**Conclusions:**

The combined model based on clinical and mpMRI findings improved csPCa prediction and might be useful in making a decision about which patient could safely avoid unnecessary SB in addition to MRTB in biopsy-naïve patients.

**Critical relevance statement:**

The combined model based on clinical and mpMRI findings improved csPCa prediction and might be useful in making a decision about which patient could safely avoid unnecessary SB in addition to MRTB in biopsy-naïve patients.

**Key points:**

• Age, PSAD, PI-RADS score, and peripheral index lesion were independent predictors of csPCa.

• Risk models were used to predict the probability of detecting csPCa on cognitive MRTB.

• The combined model might reduce 43.8% of unnecessary SBs, while missing 2.9% csPCa.

**Graphical Abstract:**

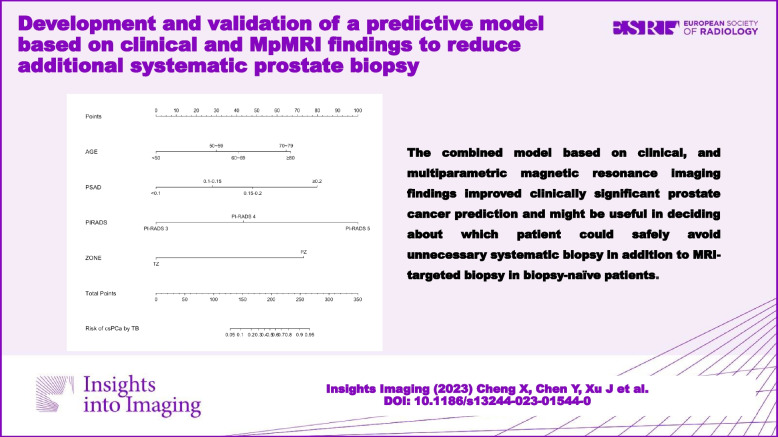

**Supplementary Information:**

The online version contains supplementary material available at 10.1186/s13244-023-01544-0.

## Introduction

Prostate cancer (PCa) is the second most common cancer and the sixth leading cause of cancer-related deaths among men worldwide [1]. Multiparametric magnetic resonance imaging (mpMRI) allows for better identification of clinically significant PCa (csPCa) and image-guided biopsy targeting suspicious lesions (i.e., prostate imaging data and reporting system, PI-RADS ≥ 3) [2]. The use of prebiopsy mpMRI as a triage test could reduce unnecessary biopsies by a quarter [3]. Meanwhile, MRI-targeted biopsy (MRTB) alone demonstrated noninferior detection rates of csPCa and decreased detection of clinically insignificant PCa (cisPCa) compared with transrectal ultrasound (TRUS)-guided systematic biopsy (SB) in biopsy-naïve men [3]. Prominent medical organizations advocate combined MRTB with SB in biopsy-naïve men with MRI suspicious lesions to minimize the incidence of missing csPCa [4, 5]. Nevertheless, the necessity of performing concurrent SB is still a matter of debate. On the one hand, the additional SB detected only a marginally more high-risk cancer but undesirable, much more cisPCa [6, 7]. On the other hand, the additional 10 or 12 systematic cores would increase patients’ pain and complication, workload of pathologists, and the cost of medical services [8]. Therefore, rather than a “one-size-fits-all” biopsy approach, optimizing the indication for performing MRTB alone thus reducing additional SB is appealed.

PI-RADS score may represent one of the indicators for avoiding additional SB among biopsy-naïve patients. It has been reported that SB marginally increases csPCa detection in patients with PI-RADS 5 on mpMRI and suggested that additional SB could be omitted in this population [9–13]. However, clinical decision-making is not based on MRI findings alone. In fact, other radiological and clinical characteristics such as index lesion features, prostate-specific antigen density (PSAD), and prostate volume (PV) may also have an impact on the added value of SB versus MRTB [14–17]. In this study, we aimed to develop and validate a novel risk model based on clinical and MRI parameters to predict the probability of detecting csPCa on cognitive MRTB. Using the predictive model, we attempt to optimize selecting patients in which omitting SB would result in a negligible risk of missing csPCa, thus reducing the number of biopsy cores.

## Methods

### Study population

This retrospective study was approved by the local Institutional Review Board (IRB), and the requirement for written consent was waived. Between May 2018 and June 2022, 355 consecutive patients who underwent combined targeted cognitive MRTB and SB were recruited. Patients were excluded for prior prostate biopsy (*n* = 47), prior prostate therapy before biopsy (*n* = 5), or prostate mpMRI not performed at our institution (*n* = 29). Finally, 274 patients were enrolled and temporally split into development (*n* = 201, from May 2018 to May 2020) and validation (*n* = 73, from June 2020 to June 2022, Fig. 1) cohorts.

**Fig. 1:**
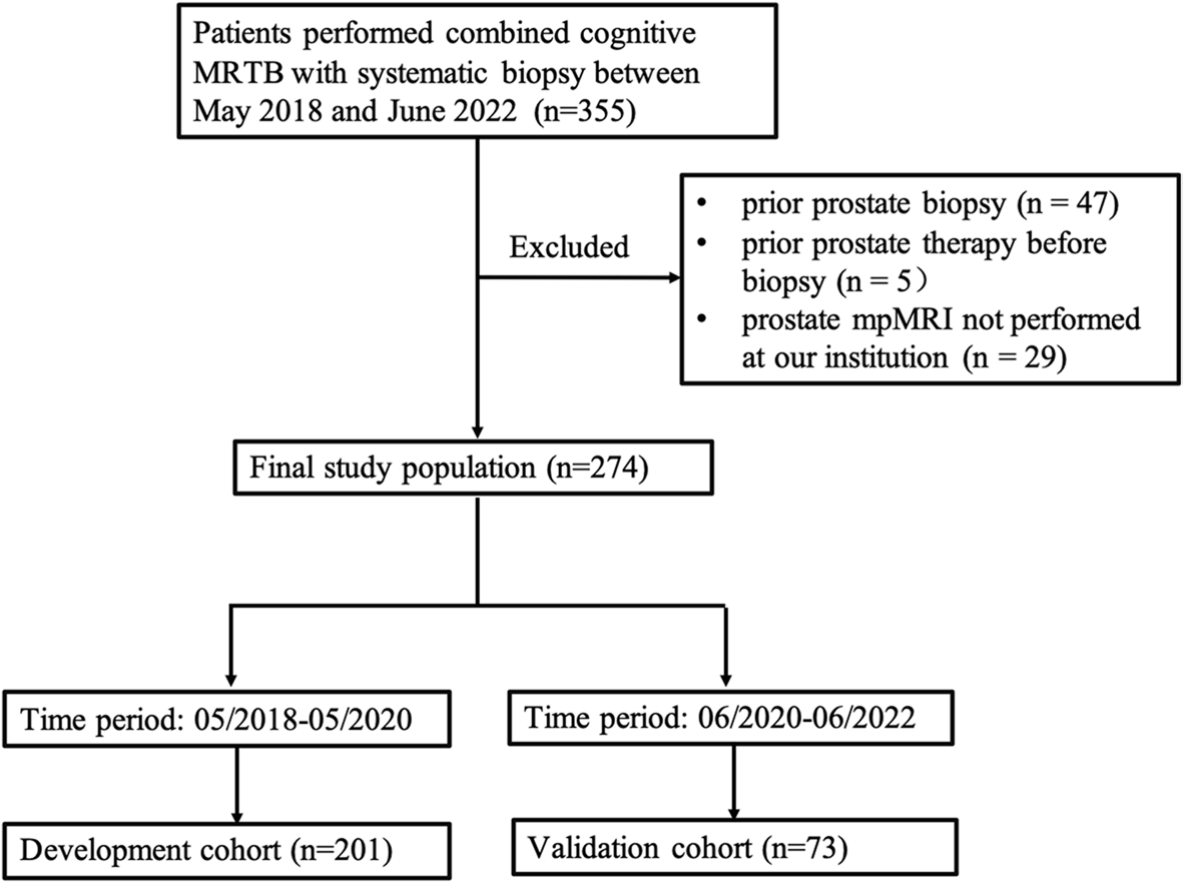
Flowchart showing the patient selection process. MRTB, MRI-target biopsy; mpMRI, multiparametric MRI

### Prostate mpMRI examination and evaluation

All patients underwent standardized prostate mpMRI using a 3.0-T MRI (Magnetom Skyra, Siemens). An 18-channel phased-array body surface coil was used. Routine prostate MRI included the following sequences [18]: (a) T2w turbo spin-echo sequences in axial—repetition time (TR), 4400 ms; echo time (TE), 96 ms; slice thickness, 3.5 mm; and matrix, 288 × 224; (b) DWI sequences—TR 3200 ms; TE, 70 ms; slice thickness, 3.5 mm; exponential *b* values, 0, 200, 800, and 1400 s/mm^2^; and the apparent diffusion coefficient (ADC) maps were calculated and constructed based on two *b* values (800 s/mm^2^ and 0 s/mm^2^); and (c) DCE MRI images were obtained using gradient-echo T1-weighted (T1w) sequences in the axial plane—TR, 3.9 ms; TE, 1.9 ms; slice thickness, 2 mm; time resolution, 12 sections/3 s; and matrix 320 × 190 after i.v. of gadolinium-based contrast material of 0.2 ml/kg of gadopentetate dimeglumine (Magnevist, Bayer Schering Pharma).

All MR images were retrospectively interpreted by two dedicated radiologists (X.C. with 3 years of experience and J.Y. with more than 5 years of experience in prostate mpMRI) following the PI-RADS v2.1 [18]. The two readers reached a consensus and determined the PI-RADS categories. PV was measured on MRI (*V* = 0.52 × transverse × anteroposterior × vertical diameter). Lesions with the highest PI-RADS score on mpMRI were defined as index lesions (IL). If there were two or more lesions with an equally high PI-RADS score, then the largest lesion was designated as the IL. Tumor focality was defined as solitary or multifocal. The maximum diameter (MD) of the IL was measured on the sequence that best defined the tumor. The sector most occupied by IL was designated as its location. The location of the IL was stratified according to the zone (peripheral or transitional zone), level (base, midgland, or apex), and orientation (anterior or posterior). A 36-region standardized prostate MRI reporting scheme was used for the visualization of mpMRI findings to the urologist.

### Prostate biopsy and pathological examination

All prostate biopsies were performed transperineally under local anesthesia. Firstly, with the MRI and diagrammatic report on a screen next to the patient, the ultrasound physician (D.C. with > 3 years of experience in targeted biopsy) scanned the prostate using transrectal ultrasonography and helped direct the urologic operator (Z.L. or a nonauthor with 3 years of experience in targeted biopsies) to aim the biopsy target at the prostate area corresponding to the lesion seen on MRI. One to five targeted biopsy cores were obtained cognitively from the suspicious lesions on MRI (PI-RADS score ≥ 3). Then, freehand TRUS-guided standard 12 cores of SB were obtained by the same urologists. All specimens were individually labeled and reviewed by two dedicated uropathologists. Gleason scores (GS) and grade groups were assigned for PCa according to the 2014 International Society of Urologic Pathology (ISUP) criteria [19]. CsPCa was defined as GS 3 + 4 or higher in at least one biopsy core.

### Variable definitions and outcomes

Clinical variables included age, prostate-specific antigen (PSA) level, PV, and PSAD. The mpMRI parameters consisted of the PI-RADS score, tumor focality, MD, zone, level, and orientation of the IL. MD was categorized into < 0.8 cm, 0.8–1.5 cm, and ≥ 1.5 cm. The number of MRTB cores consists of biopsy parameters. The outcome of interest was the detection of csPCa by cognitive MRTB.

### Statistical analysis

The *t* test, Wilcoxon test, and chi-squared test were used to compare continuous and categorical variables. The detection of csPCa was analyzed on a per-patient level. First, we performed univariate logistic regression analyses of all variables to predict csPCa detection by MRTB. Pearson’s correlation matrix was used to assess multicollinearity between age, PSA, PV, and PSAD. Secondly, multivariate logistic regression analyses (MVA) were used to identify predictors of csPCa diagnosed at MRTB. There were three models constructed: (i) the clinical model using clinical predictors, (ii) the MRI model using mpMRI predictors, and (iii) the combined model incorporating both independent clinical and mpMRI predictors. Reference strategies were avoiding or performing additional SB in all biopsy-naïve patients. The discrimination of models was assessed by the area under the receiver operating characteristic curve (AUC), and AUC differences were evaluated using the DeLong test. A calibration plot was constructed to assess the model calibration, which refers to the agreement between the observed endpoints and predictions. Moreover, decision curve analysis (DCA) was used to determine the net clinical benefit associated with the use of the models. A nomogram was developed for the model with the best predictive performance. All statistical tests were performed using SPSS version 25.0 (IBM Corp) and R statistical package v.3.0.2 (R Project for Statistical Computing, www.r-project.org). All tests were two-sided, with the significance level set at *p* < 0.05.

## Results

### Patient demographics

Patient characteristics including baseline clinical parameters, MRI, and biopsy results are summarized in Table 1. The prevalence of csPCa was 49.5% (100/201) and 46.6% (34/73) in the development and validation cohorts, respectively. Cognitive MRTB alone would have missed four cases of csPCa and underestimated one case of csPCa with GS 3 + 4 as GS 3 + 3 in the development cohort (four of them scored PI-RADS 4 and one patient scored PI-RADS 5) and would have missed four cases of csPCa in the validation cohort (one of them scored PI-RADS 5, one patient scored PI-RADS 4, and two patients scored PI-RADS 3). The variables between cohorts were not significantly different, except for the targeted biopsy cores per lesion (Table 1).

**Table 1:** Patient demographics including baseline clinical parameters, MRI, and biopsy results of the development and validation cohorts

Variable	Development cohort (*n* = 201)	Validation cohort (*n* = 73)	*p*
No. of patients	201	73	–
Time period (month/year)	05/2018 to 05/2020	06/2020 to 06/2022	
Age (years)	66.1 ± 9.16	67.3 ± 8.44	0.344
PSA (ng/ml)	10.3 (6.5–14.9)	12.4 (7.4–16.5)	0.063
Volume (ml)	37.2 (29.1–54.0)	43.4 (30.5–62.2)	0.189
PSAD (ng/ml^2^)	0.24 (0.16–0.42)	0.28 (0.17–0.39)	0.53
Diameter (mm)	1.4 (1.1–1.8)	1.4 (1.0–1.8)	0.226
Targeted cores per lesion	3.5 ± 1.33	2.7 ± 1.10	< 0.001
Biopsy results			
No cancer	87 (43.3%)	33 (45.2%)	0.873
GS 3 + 3	14 (7.0%)	6 (8.2%)	
GS ≥ 3 + 4	100 (49.5%)	34 (46.6%)	
PI-RADS score			
3	52	25	0.387
4	91	30	
5	58	18	
Solitary/multifocal	128/73	44/29	0.672
Orientation			
Anterior	112	42	0.891
Posterior	89	31	
Level			
Base	41	16	0.959
Mid	105	37	
Apex	55	20	
Zone			
Peripheral	86	37	0.273
Transitional	115	36	

*GS* Gleason score, *PI-RADS* Prostate Imaging Reporting and Data System, *PSA* prostate-specific antigen, *PSAD* prostate-specific antigen density

### Development and validation of multivariable logistic regression models

In the univariate analysis, age (*p* = 0.008), PSA level (*p* = 0.003), PV (*p* < 0.001), PSAD (*p* < 0.001), PI-RADS score (*p* < 0.001), MD (*p* = 0.028), zone (*p* < 0.001), and IL level (*p* < 0.001) were significant as single explanatory predictors of csPCa detection by MRTB. Tumor focality (*p* = 0.18), IL orientation (*p* = 0.161), and number of targeted cores per lesion (*p* = 0.19) were not predictive of csPCa detection by MRTB (all *p* > 0.05). Pearson’s correlation matrix showed that PSAD was strongly associated with PSA levels (*ρ* = 0.78) and PV (*ρ* =  − 0.59). To avoid multicollinearity, age (odds ratio [OR] 1.63, 95% confidence interval [CI] 1.15–2.30; *p* = 0.006) and PSAD (OR 2.72, 95% CI 1.86–3.98; *p* < 0.001) were included in the clinical model because PSAD is a stronger predictor than PSA level and PV [20]. At MVA, PI-RADS score (PI-RADS 4 OR 4.24, 95% CI 1.48–12.15; PI-RADS 5 OR 50.13, 95% CI 12.67–198.32; *p* < 0.001), IL on the peripheral zone (OR 6.52, 95% CI 2.83–14.99; *p* < 0.001), and IL on the apex level (OR 3.48, 95% CI 1.09–11.04; *p* = 0.035) resulted to be significantly associated with the probability of detecting csPCa at cognitive MRTB (Table 2) and incorporated the MRI model. Furthermore, age (OR 2.3, 95% CI 1.42–3.72; *p* = 0.001), PSAD (OR 2.3, 95% CI 1.44–3.65; *p* < 0.001), PI-RADS score (PI-RADS 4 OR 5.17, 95% CI 1.69–15.72; PI-RADS 5 OR 51.58, 95% CI 11.78–225.86; *p* < 0.001), and IL on peripheral zone (OR 9.35, 95% CI 3.56–24.56; *p* < 0.001) remained independent predictor status (Table 2) and incorporated the combined model.

**Table 2:** Multivariate logistic regression model analysis for the prediction of detecting csPCa by cognitive MRTB in biopsy-naive men

Characteristic	Level	Clinical model			MRI model			Advanced model		
		**Coefficient**	**OR (95% CI)**	***p***	**Coefficient**	**OR (95% CI)**	***p***	**Coefficient**	**OR (95% CI)**	***p***
Intercept		− 5.05	–	< 0.001	− 3.51	–	0.001	–8.38	–	< 0.001
Age (years)	+ 10	0.487	1.63 (1.15, 2.30)	0.006	–	–	–	0.83	2.30 (1.42, 3.72)	0.001
PSAD (ng/ml^2^)	+ 0.05	1.0	2.72 (1.86, 3.98)	< 0.001	–	–	–	0.83	2.30 (1.44, 3.65)	< 0.001
PI-RADS score				–			< 0.001			< 0.001
	3	–	–	–		1	–		1	–
	4	–	–	–	1.44	4.24 (1.48, 12.15)	0.007	1.64	5.17 (1.69, 15.72)	0.004
	5	–	–	–	3.92	50.13 (12.67, 198.32)	< 0.001	3.94	51.58 (11.78, 225.86)	< 0.001
Zone of IL	TZ		–	–		1			1	–
	PZ	–	–	–	1.87	6.52 (2.83, 14.99)	< 0.001	2.34	9.35 (3.56, 24.56)	< 0.001
Level of IL				–			0.107			0.563
	Base	–	–	–		1			1	–
	Mid	–	–	–	0.76	2.14 (0.76, 6.02)	0.149	0.28	1.32 (0.42, 4.14)	0.635
	Apex	–	–	–	1.25	3.48 (1.09, 11.04)	0.035	0.67	1.95 (0.54, 7.04)	0.307
MD of IL (cm)				–			0.831	–	–	–
	< 0.8	–	–	–		1	–	–	–	–
	0.8–1.4	–	–	–	0.112	1.12 (0.19, 6.54)	0.901	–	–	–
	≥ 1.5	–	–	–	–0.19	0.827 (0.124, 5.50)	0.845	–	–	–

*CI* confidential interval, *IL* index lesion, *MD* maximum diameter, *OR* odds ratio, *PI-RADS* Prostate Imaging Reporting and Data System, *PSAD* prostate-specific antigen density

**Fig. 2:**
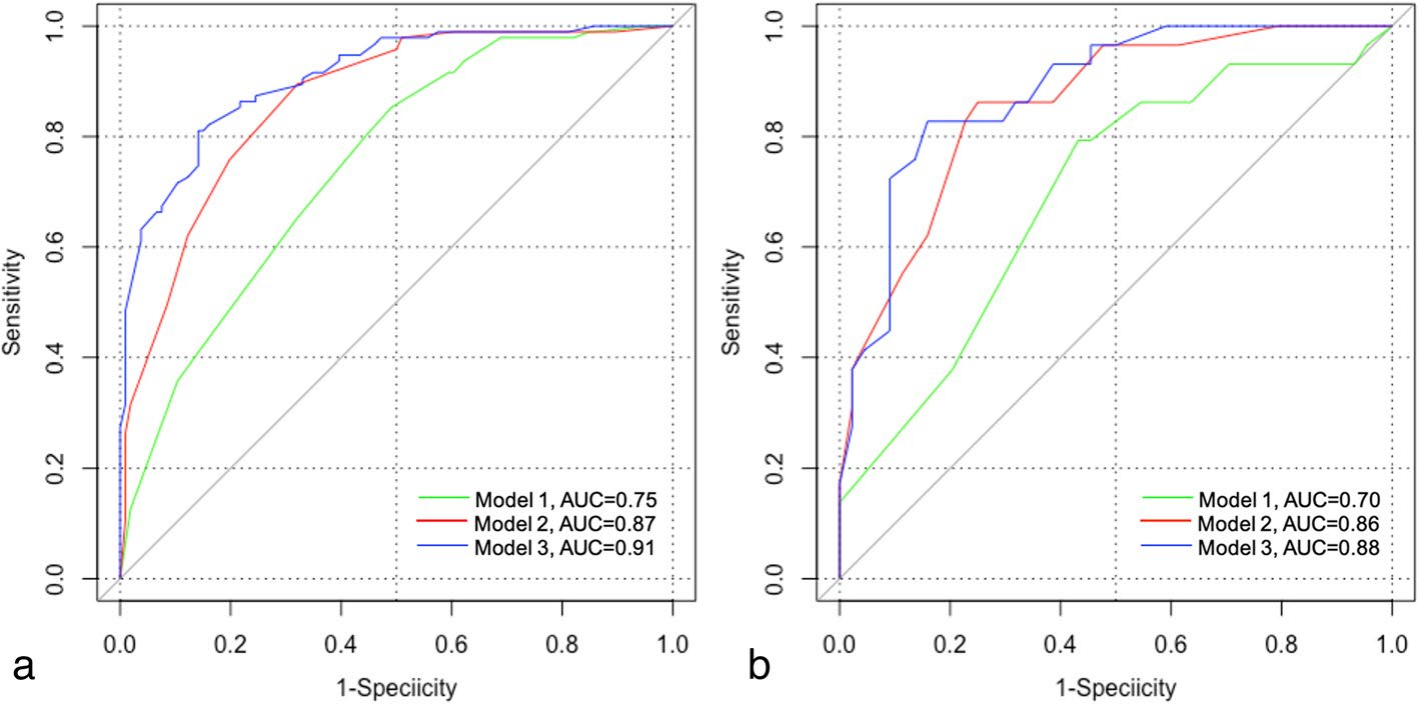
Receiver operating characteristic (ROC) curves of the risk prediction models for the development (**a**) and validation (**b**) cohorts. Model 1 = clinical model, model 2 = MRI model, and model 3 = combined model. AUC, area under the ROC curve

**Fig. 3:**
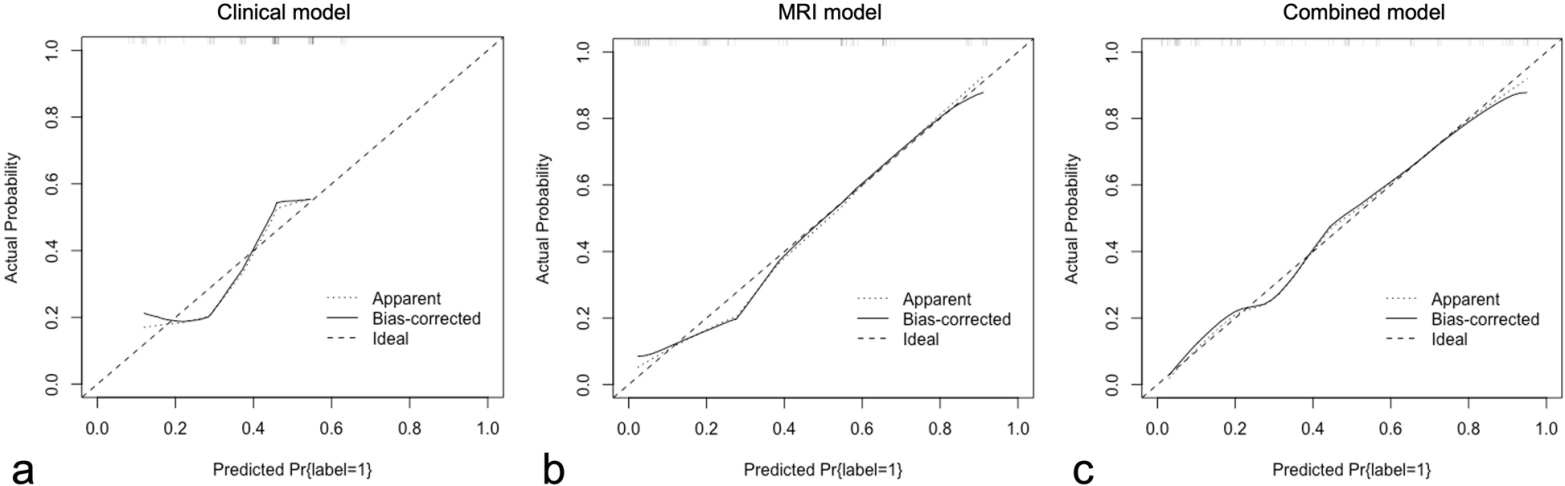
Calibration plots of the risk prediction models when applied to the validation cohort. **a** Clinical model. **b** MRI model. **c** Combined model

**Fig. 4:**
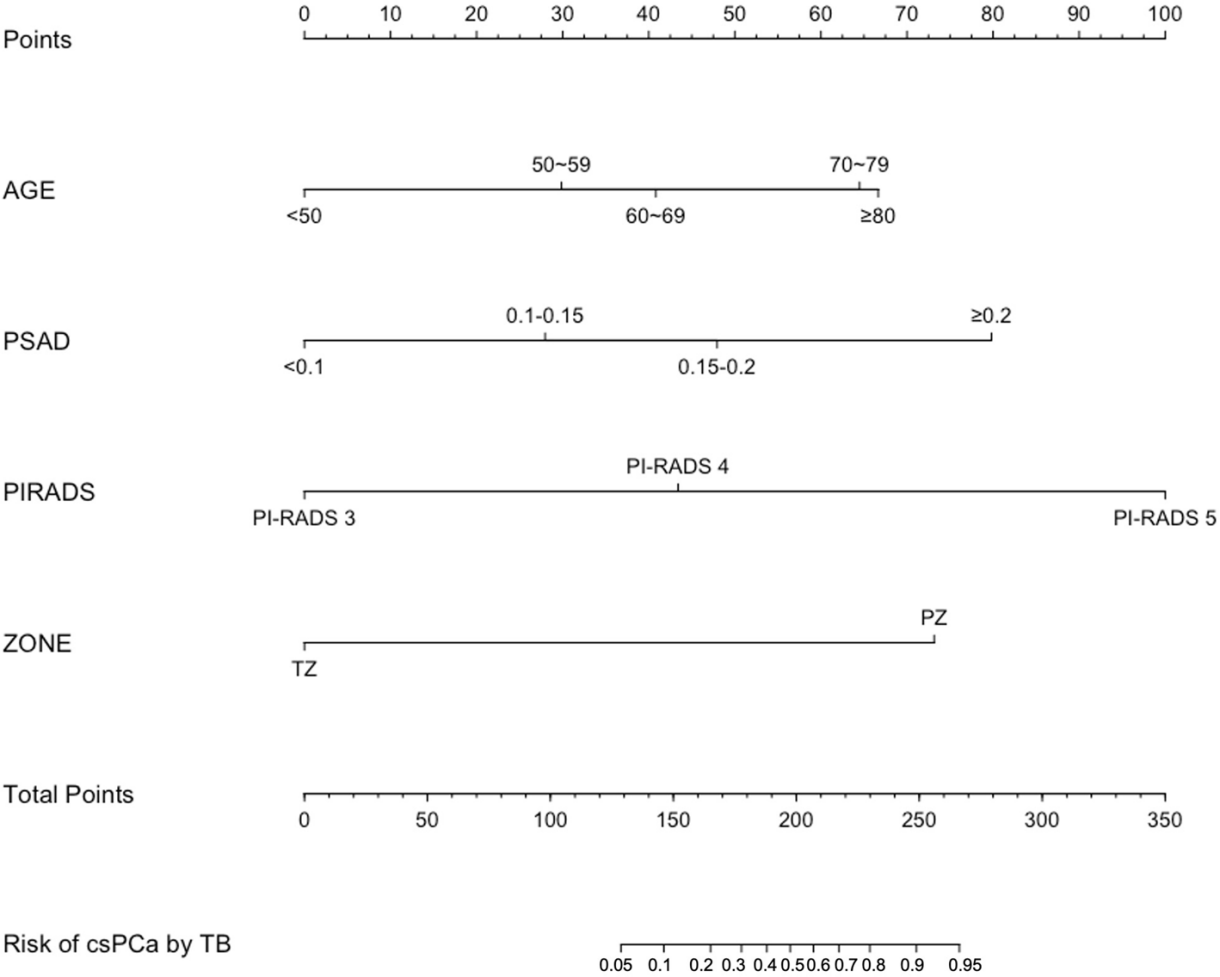
Nomogram combined model. Nomogram predicting the probability of detecting csPCa by cognitive MRTB for biopsy-naïve patients. PSAD, prostate-specific antigen density; PI-RADS, Prostate Imaging Reporting and Data System; TZ, transitional zone; PZ, peripheral zone

The MRI model achieved a greater AUC than the clinical model in both the development (AUC 0.87, 95% CI 0.82–0.91 vs. AUC 0.75, 95% CI 0.68–0.81; *p* = 0.006) and validation (AUC 0.86, 95% CI 0.77–0.94 vs. AUC 0.70, 95% CI 0.58–0.82; *p* = 0.038) cohorts. Compared with the clinical model, the combined model achieved an increase in AUC from 0.75 to 0.91 (95% CI 0.87–0.95, *p* < 0.001) and from 0.70 to 0.88 (95% CI 0.81–0.96, *p* = 0.001) in the development and validation cohorts, respectively (Fig. 2). However, the combined model achieved a slight increase in AUC compared to the MRI model (0.88 vs. 0.86, *p* = 0.43) in the validation cohort. As shown in Additional file 1: Fig. S1, the three models exhibited good agreement between the observed and predicted outcomes in the development cohort. In the validation cohort, the calibration plot demonstrated a superior fit of the combined model compared with the other two models (Fig. 3). Therefore, the combined model was selected as the best-performing prediction model, and a nomogram was derived from it (Fig. 4).

### Decision curve analysis

**Fig. 5:**
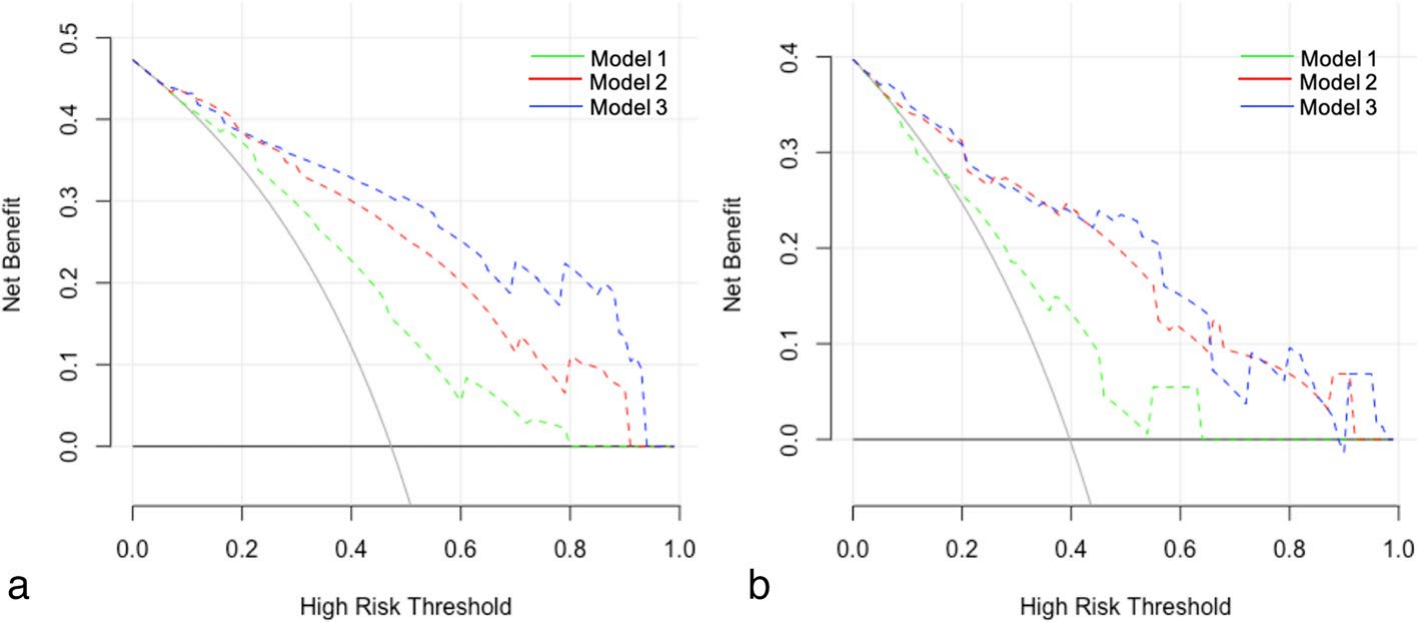
Decision curves of the risk prediction models for the development (**a**) and validation cohorts (**b**). Model 1 = clinical model, model 2 = MRI model, and model 3 = combined model. The risk thresholds range from 0 to 100%

In the development cohort, all three risk prediction models enabled higher net benefits than those of the “treat all” (avoid additional SB in all suspected patients) and “treat none” (perform concurrent SB in all suspected patients) approaches at risk thresholds between 10 and 80% (Fig. 5a). Meanwhile, the net benefits of the combined model outperformed those of the other two models at risk thresholds between 20 and 90%. In the validation cohort, the combined model enabled the highest net benefit at a risk threshold between 50 and 65% (Fig. 5b). A systematic analysis of the net benefits of the risk prediction models at a risk threshold between 50 and 90% is presented in Additional file 1: Table S1. Decision curves for the entire range of risk thresholds (50–100%) are shown in Additional file 1: Fig. S2.

**Fig. 6:**
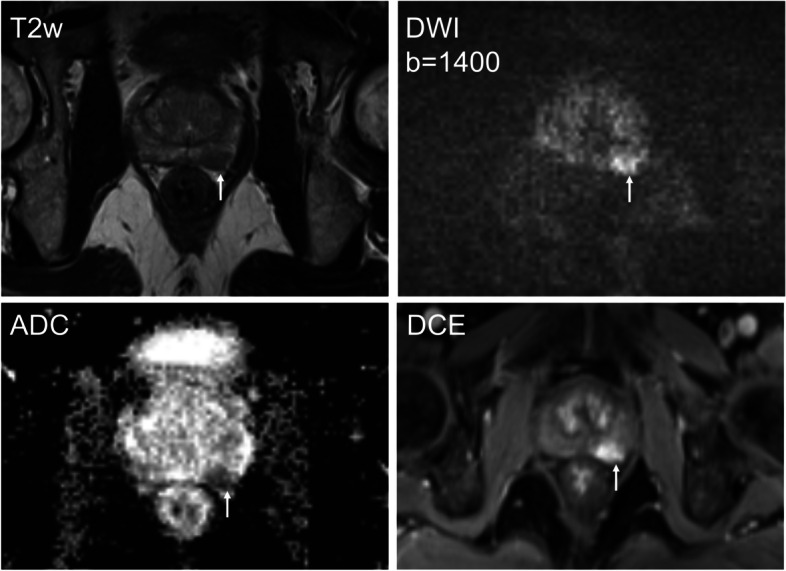
MR images in a 63-year-old man with a prostate-specific antigen (PSA) level of 7.39 ng/ml. The prostate volume measured by MRI was 42.42 ml, and the PSA density was 0.174 ng/ml^2^. Multiparametric MRI demonstrated a lesion measuring 1.1 cm in the left posterior peripheral zone midgland (arrow) and ill-defined margins on T2-weighted images with severe restricted diffusion and early contrast enhancement; Prostate Imaging Reporting and Data System (PI-RADS) category 4. On subsequent cognitive MRI-target biopsy, all 3 targeted cores demonstrated Gleason score (GS) 4 + 5 with maximum tumor core involvement of 57%. Five out of 12 systematic biopsy (SB) cores were positive for prostate cancer with the highest GS 4 + 5 and maximum tumor core involvement of 26%. At a risk threshold of 60%, both the MRI model (predicted risk: 67.6%) and the combined model (predicted risk: 65.6%) would have resulted in obviating SB. The predicted risk for the clinical model was 35.7%. The patient would have undergone unnecessary SB based on the clinical model and PI-RADS 5 strategy. ADC, apparent diffusion coefficient; DWI, diffusion-weighted imaging; DCE, dynamic contrast-enhanced; T2w, T2-weighted imaging

Table 3 shows the absolute number of SBs reduced versus csPCa detected and missed by MRTB using different biopsy strategies. At a risk threshold of 60%, 88 of 201 (43.8%) and 32 of 73 (43.8%) patients were advised to avoid additional SB, leaving one of 100 (1%) and one of 34 (2.9%) patients with csPCa missed using the combined model in the development and validation cohorts, respectively. Conversely, performing MRTB only in men with PI-RADS category 5 lesions would have reduced unnecessary SBs in 58 of 201 (28.9%) patients and 18 of 73 (24.7%) patients, leaving 2 of 100 (2%) and 1 of 34 (2.9%) patients with csPCa missed in the development and validation cohorts, respectively. A representative case with mpMRI and the biopsy outcome is shown in Fig. 6.

**Table 3:** Systematic biopsies reduced versus csPCa detected and/or missed by cognitive MRTB in the development and validation cohorts using different biopsy strategies

Threshold	Models	The development cohort (*n* = 201)				The validation cohort (*n* = 73)			
		**No. of SB performed**	**No. of SB reduced**	**No. csPCa detected by MRTB**	**No. csPCa missed by MRTB**	**No. of SB performed**	**No. of SB reduced**	**No. csPCa detected by MRTB**	**No. csPCa missed by MRTB**
50%	Clinical	105 (52.2%)	96 (47.8%)	99 (99%)	1 (1%)	53 (72.6%)	20 (27.4%)	32 (94.1%)	2 (5.9%)
	MRI	98 (48.8%)	103 (51.2%)	98 (98%)	2 (2%)	39 (53.4%)	34 (46.6%)	33 (97.1%)	1 (2.9%)
	Advanced	106 (52.7%)	95 (47.3%)	98 (98%)	2 (2%)	41 (56.2%)	32	33 (97.1%)	1 (2.9%)
60%	Clinical	105 (52.2%)	96 (47.8%)	99 (99%)	1 (1%)	53 (72.6%)	20 (27.4%)	32 (94.1%)	2 (5.9%)
	MRI	108 (53.7%)	93 (46.3%)	98 (98%)	2 (2%)	39 (53.4%)	34 (46.6%)	33 (97.1%)	1 (2.9%)
	Advanced	113 (56.2%)	88 (43.8%)	99 (99%)	1 (1%)	41 (56.2%)	32 (43.8%)	33 (97.1%)	1 (2.9%)
70%	Clinical	156 (77.6%)	45 (22.4%)	99 (99%)	1 (1%)	53 (72.6%)	20 (27.4%)	32 (94.1%)	2 (5.9%)
	MRI	129 (64.2%)	72 (35.8%)	98 (98%)	2 (2%)	39 (53.4%)	34 (46.6%)	33 (97.1%)	1 (2.9%)
	Advanced	130 (64.7%)	71 (35.3%)	99 (99%)	1 (1%)	48 (65.8%)	25 (34.2%)	33 (97.1%)	1 (2.9%)
80%	Clinical	187 (93.0%)	14 (7.0%)	100 (100%)	0 (0%)	53 (72.6%)	20 (27.4%)	32 (94.1%)	2 (5.9%)
	MRI	175 (87.1%)	26 (12.9%)	100 (100%)	0 (0%)	48 (65.8%)	25 (34.2%)	33 (97.1%)	1 (2.9%)
	Advanced	137 (68.2%)	64 (31.8%)	99 (99%)	1 (1%)	48 (65.8%)	25 (34.2%)	33 (97.1%)	1 (2.9%)
90%	Clinical	201 (100%)	0 (0%)	100 (100%)	0 (0%)	53 (72.6%)	20 (27.4%)	32 (94.1%)	2 (5.9%)
	MRI	177 (88.1%)	24 (11.9%)	100 (100%)	0 (0%)	52 (71.2%)	21 (28.8%)	33 (97.1%)	1 (2.9%)
	Advanced	164 (81.6%)	37 (18.4%)	100 (100%)	0 (0%)	49 (67.1%)	24 (32.9%)	33 (97.1%)	1 (2.9%)
–	PI-RADS 5	143 (71.1%)	58 (28.9%)	98 (98%)	2 (2%)	55 (75.3%)	18 (24.7%)	33 (97.1%)	1 (2.9%)

*Note*: Data in percentages are percentages of all performed additional systematic biopsies or all clinically significant prostate cancers (csPCas) detected by cognitive MRI-target biopsy

*PI-RADS* Prostate Imaging Reporting and Data System, *MRTB* MRI-targeted biopsy, *SB* systematic biopsy

## Discussion

Recently, upfront mpMRI has been utilized as a triage test for men with low-suspicion MRI to avoid biopsy, while those with suspicious lesions on MRI would undergo only MRTB [21]. However, clinical decision-making cannot be based on MRI findings alone. Clinical features, such as PSA, PSAD, ethnicity, family history of PCa, biopsy history, and digital rectal examination findings, are also important for decision-making. In this study, we developed and validated a combined model that incorporated both clinical and mpMRI characteristics to predict the probability of detecting csPCa by cognitive MRTB. We found that the combined model achieved the best discrimination compared with both the clinical model including age and PSAD, and the MRI model including PI-RADS score, zone, and level of IL. The models were well calibrated in internal and external validation, with decision analysis showing that the use of the combined model in practice would improve clinical decision-making about avoiding additional SBs in biopsy-naïve men.

MRTB was reported to be superior to SB in detecting csPCa in older patients (> 50 years) [22]. A higher MRI suspicion level (analogous to PI-RADS 4 and 5) was associated with the detection rate of csPCa or high-grade PCa by MRTB [23]. Kuhlmann et al. found that an anterior or apical lesion location favors better PCa capture on targeted biopsies [24]. Wibulpolprasert et al. described a significantly greater sensitivity of mpMRI for peripheral zone tumors than for transitional zone tumors [25]. In the present study, age, PSAD, PI-RADS score, and IL in the peripheral zone were independent predictors of csPCa on cognitive MRTB, and a combined predictive model was developed accordingly. It had significantly superior discrimination compared with the clinical model but only a slight increase in the AUC compared with the MRI model in our validation cohort. A larger number of patients included in the validation cohort would be expected to achieve an even greater benefit in favor of the combined model.

Our study design allows us to quantify model robustness to temporal dynamics using a temporal data split. However, temporal heterogeneity has been reported to affect the calibration and clinical applicability of risk-prediction models [26]. In the validation cohort, the combined model was best calibrated but did not always have the best clinical utility. At the 50–65% cutoff, the combined model was superior to both the clinical and MRI models in terms of net benefit. However, the DCA curve of the combined model drifted, and its net benefit was sometimes inferior to that of the MRI model beyond this threshold range. Reasons from the urologist selecting patients for biopsy to improving technical standards of prostate mpMRI and increasing expertise in the interpretation of prostate mpMRI and targeted biopsy techniques may result in temporal performance drifts of prediction models [27].

Currently, not all men benefit from SB biopsy in addition to MRTB because the additional 10–12 cores may certainly increase costs, patient pain, and complications [9], as mentioned previously. An increasing number of recent studies have suggested that additional SB could be omitted in select patients such as men with PI-RADS 5 lesions [9–13]. In Deniffel et al.’s study, a biopsy strategy where additional SB is omitted in men with PI-RADS 5 and/or prior negative biopsy would avoid excess biopsy in 58% and cisPCa diagnosis in 3% of men while missing csPCa in only 2% [11]. Other studies aimed to explore the different clinical and mpMRI parameters. Tafuri et al. reported that in the subgroup of patients with PI-RADS score 5 and PSAD > 0.15, the added value of SB to software registration fusion MRTB in detection csPCa was only 4% [10]. Barletta et al. focused on the volume of IL at mpMRI and found that only 15% of men with PI-RADS 3 lesions and volume > 1.2 ml and 29% of men with PI-RADS 4 lesions and volume > 0.6 ml obtained a marginal benefit from the addition of SB in terms of csPCa detection (~ 4%) [28]. Although in-bore or software registration fusion would be an ideal approach of MRTB for allowing better visualization and sampling of smaller lesions, cognitive fusion targeting implemented with significant experience could achieve similar accuracy in csPCa detection [29]. Meanwhile, the main meta-analysis also failed to demonstrate a significant advantage of any MRTB technique over others with regard to csPCa detection [30]. Along with those previous studies using software fusion MRTB, our pilot retrospective study using cognitive targeting also demonstrated that additional SB could be safely avoided in selected patients in terms of diagnostic purposes [10, 28]. In the present study, the strategy of performing cognitive MRTB alone in PI-RADS 5 biopsy-naïve patients would result in a 28% and 24.7% reduction in unnecessary SBs, while missing 2% and 2.9% csPCa in the development and validation cohorts, respectively. Besides, the combined model incorporated both clinical and mpMRI characteristics achieved greater net benefit and would help clinical decision-making comprehensively. With a risk threshold set at 60%, 43.8% of patients with very high risk (greater than 60%) of detecting csPCa on MRTB would be advised to avoid SB both in the development and validation cohorts. Accordingly, a total of 1440 systematic cores would be saved at the expense of missing only two cases of csPCa.

The findings from this study and the literature support the premise of performing MRTB alone in selected men. However, it remains critical to relate these risks to the patient’s preferences and willingness to compromise between the decreased cost, pain, and complications due to reduced biopsy cores and the potential risk of missing csPCa because of inaccurate targeted sampling. This may result in the need for repeated prostate biopsies. Meanwhile, the optimal patient selection strategies should consider a clinical scenario of specific treatment approaches (e.g., focal therapy or operative planning), where additional SB may implicit the presence of multifocal PCa [9, 31]. Other studies that argue in favor of additional SB have suggested a complementary prognostic role of SB for adverse pathological features and prognosis after treatment (e.g., extracapsular extension, biochemical recurrence) [32, 33] but are limited by the fact that they did not consider PI-RADS scores, one of the main predictors of PCa aggressiveness [11].

The present study had several limitations. Firstly, we just focused on the primary outcome of detection of csPCa but did not consider SB added value in terms of disease upgrading on radical prostatectomy. Therefore, our results should be considered for diagnostic purposes only. We acknowledge that SBs still play an important role in patients’ risk assessment and in some specific clinical scenarios. Secondly, the predictive models were contemporarily validated in the same hospital with a small sample size; thus, their performance remains unclear when applied to other clinical centers. Further prospective multicenter validation is justified to test our observations. Thirdly, cognitive confusion instead of software-based co-registration MRTB was performed in the study, even though the operators had significant clinical experience. Hence, the results should be cautiously considered and might not be generalizable to other cohorts.

## Conclusion

In this study, we developed a novel risk model that integrates clinical and mpMRI findings including age, PSAD, PI-RADS score, and IL zone to predict the probability of detecting csPCa by cognitive MRTB. The predictive model might be useful in making a decision about which patient could safely avoid unnecessary SB in addition to MRTB in biopsy-naïve patients and may offer the best compromise between the risk of missing csPCa, biopsy complications, and medical burden. Further perspective and multicenter study are required to validate the model’s performance and applicability.

### Supplementary Information

**Additional file 1:** **Fig. S1.** Calibration plots of risk prediction models when applied to the development cohort: (A) clinical model, (B) MRI model, (C) combined model. **Fig. S2.** Decision curves of risk prediction models for development cohort (A) and validation cohort (B). Model 1=clinical model, Model 2=MRI model, Model 3=combined model. The risk thresholds range from 50%-100%. **Table S1.** Net benefits of the clinical model, the MRI model and the combined model determined in the development cohort and the validation cohort using decision curve analysis.

## Data Availability

The datasets used and/or analyzed during the current study are available from the corresponding authors upon reasonable request.

## Abbreviations

AUC: Area under receiver operator characteristic curve
CI: Confidence interval
cisPCa: Clinically insignificant prostate cancer
csPCa: Clinically significant prostate cancer
DCA: Decision cure analysis
GS: Gleason score
IL: Index lesion
ISUP: International Society of Urologic Pathology
MD: Maximum diameter
MRTB: MRI-targeted biopsy
mpMRI: Multiparametric magnetic resonance imaging
PCa: Prostate cancer
PI-RADS: Prostate Imaging-Reporting and Data System
PSA: Prostate-specific antigen
PSAD: Prostate-specific antigen density
PV: Prostate volume
ROC: Receiver operator characteristic curve
SB: Systematic biopsy
TRUS: Transrectal ultrasound
