# Supplementary material for: Development and validation of a predictive model based on clinical and MpMRI findings to reduce additional systematic prostate biopsy
Source: Insights Imaging. 2024 Jan 7;15:3. doi: 10.1186/s13244-023-01544-0 (PMC10772021; doi:10.1186/s13244-023-01544-0)
Supplement: Supplementary file 1 — Additional file 1: Fig. S1. Calibration plots of risk prediction models when applied to the development cohort: (A) clinical model, (B) MRI model, (C) combined model. Fig. S2. Decision curves of risk prediction models for development cohort (A) and validation cohort (B). Model 1=clinical model, Model 2=MRI model, Model 3=combined model. The risk thresholds range from 50%-100%. Table S1. Net benefits of the clinical model, the MRI model and the combined model determined in the development cohort and the validation cohort using decision curve analysis. [file 13244_2023_1544_MOESM1_ESM.docx]

**Development and validation of a Predictive Model Based on Clinical and MpMRI Findings to Reduce Additional Systematic Prostate Biopsy**

**ELECTRONIC SUPPLEMENTARY MATERIAL**

**Figure S1.** Calibration plots of risk prediction models when applied to the development cohort: (A) clinical model, (B) MRI model, (C) combined model.

**Figure S2.** Decision curves of risk prediction models for development cohort (A) and validation cohort (B). Model 1=clinical model, Model 2=MRI model, Model 3=combined model. The risk thresholds range from 50%-100%.

**Table S1.** Net benefits of the clinical model, the MRI model and the combined model determined in the development cohort and the validation cohort using decision curve analysis.

| **Threshold** | **Cost: benefit ratio** | **Treat none** | **The Development cohort (n = 201)** | | | | | **The Validation cohort (n = 73)** | | | |
| --- | --- | --- | --- | --- | --- | --- | --- | --- | --- | --- | --- |
|  |  |  | **Treat all** | **Clinical model** | **MRI model** | **Combined model** |  | **Treat all** | **Clinical model** | **MRI model** | **Combined model** |
| 50% | 1:1 | 0 | -11.6% | 29.5% | 53.7% | 64.2% |  | -51.2% | 6.9% | 48.3% | 58.6% |
| 55% | 11:9 | 0 | -36.4% | 21.5% | 48.8% | 60.2% |  | -85.4% | 13.8% | 40.6% | 52.1% |
| 60% | 3:2 | 0 | -67.4% | 11.6% | 42.6% | 53.2% |  | -127.6% | 13.8% | 29.3% | 37.9% |
| 65% | 13:7 | 0 | -107.2% | 14.3% | 34.7% | 45.4% |  | -181.8% | 0 | 23.4% | 33% |
| 70% | 7:3 | 0 | -160.4% | 8.8% | 24.2% | 47.7% |  | -254% | 0 | 23% | 12.6% |
| 75% | 3:1 | 0 | -234.7% | 6.3% | 21.1% | 41.1% |  | -355% | 0 | 20.7% | 20.7% |
| 80% | 4:1 | 0 | -346.3% | 0 | 23.2% | 46.3% |  | -506.9% | 0 | 17.2% | 24.1% |
| 85% | 17:3 | 0 | -532.3% | 0 | 20.4% | 39.3% |  | -759.8% | 0 | 11.5% | 11.5% |
| 90% | 9:1 | 0 | -904.2% | 0 | 14.7% | 28.4% |  | -1265% | 0 | 17.2% | 3.4% |
